# *Drosophila ezoana* uses morning and evening oscillators to adjust its rhythmic activity to different daylengths but only the morning oscillator to measure night length for photoperiodic responses

**DOI:** 10.1007/s00359-023-01646-6

**Published:** 2023-06-17

**Authors:** Koustubh M. Vaze, Giulia Manoli, Charlotte Helfrich-Förster

**Affiliations:** 1 Life Sciences Institute, University of Michigan, Ann Arbor, MI 48109 USA; 2 Neurobiology and Genetics, University of Würzburg, Biocentre, Theodor-Boveri-Institute, Am Hubland, 97074 Würzburg, Germany

**Keywords:** Photoperiodism, Time measurement, Circadian clock, External and internal coincidence, Fruit flies

## Abstract

Animals living at high latitudes are exposed to prominent seasonal changes to which they need to adapt to survive. By applying Zeitgeber cycles of different periods and photoperiods we show here that high-latitude *D. ezoana* flies possess evening oscillators and highly damped morning oscillators that help them adapting their activity rhythms to long photoperiods. In addition, the damped morning oscillators are involved in timing diapause. The flies measure night length and use external coincidence for timing diapause. We discuss the clock protein TIMELESS (d-TIM) as the molecular correlate and the small ventrolateral clock neurons (s-LN_v_s) as the anatomical correlates of the components measuring night length.

**Supplementary Information:**

The online version contains supplementary material available at 10.1007/s00359-023-01646-6.

## Introduction

Animals inhabiting high latitudes are adapted to cope with extreme seasonal changes. These annual changes are especially harsh in latitudes close to arctic regions, where there are extreme differences in light and temperature conditions throughout the year. Amid the different *Drosophilidae*, members of the *virilis* group, such as *Drosophila montana, D. ezoana* and *D. littoralis* are some of the best-adapted species to such conditions. They can extend their activity throughout the day even at very long day lengths of 20 h, and they retain some circadian rhythmicity even at constant light (the polar day) (Kauranen et al. 2012; Menegazzi et al. 2017; Beauchamp et al. 2018). Furthermore, they have a strong photoperiodically controlled diapause, which helps them to anticipate and survive the winter. Diapause is characterized by reproductive arrest, reduced metabolism, increased stress resistance, and prolonged lifespan, and it is initiated when the days become shorter in late summer. *D. ezoana* and *D. littoralis* enter adult diapause already at a critical night length of 7 h (7 h) at temperatures around 17 °C (Lumme et al. 1974; Vaze and Helfrich-Förster 2016). This means that they enter diapause when the days become shorter than 17 h even if the environmental temperatures are still pleasant. Just for comparison: photoperiods of 17 h are very long and never reached at latitudes below 50 °N in the northern hemispheres such as the Mediterranean regions, the Middle East, China or the United States of America (except of Alaska). Flies living at lower latitudes, e. g. *Drosophila melanogaster,* often need a combination of adverse conditions like low temperatures combined with food shortage and short photoperiods to enter a state of reproductive dormancy (Saunders et al. 1989; Kubrak et al. 2014; Ojima et al. 2018; Nagy et al. 2019). Dormancy in these species is also not strongly dependent on night/day length and can be terminated as soon as the environmental conditions improve (Saunders et al. 1989; Kostál 2006).

The strongly photoperiodic diapause of *D. ezoana* requires the measurement of night/day length. To judge the length of a night/day, an internal reference timer appears necessary. As hypothesized by Bünning (1936), the circadian clock is well suited for this role and its importance for photoperiodic responses has been demonstrated for several insects living at high latitudes (reviewed in Saunders 2020; Goto 2022; Denlinger 2023). However, there is still debate about the general principles of daylength measurement and the extent to which the circadian clock is involved (Bradshaw and Holzapfel 2010; Bradshaw et al. this issue). A fundamental problem is that the photoperiodic timer (the critical daylength that determines the onset of diapause) has evolved independently of the circadian clock (Emerson et al. 2009; Lankinen et al. 2021). Thus, a simple ‘interval timer’ or ‘hour-glass clock’ as suggested for aphids (Lees 1960, 1966) would do it as well (or even better). In the aphid *Megoura viciae*, this interval timer is reset every night by lights-off and is thought to lead to the accumulation of an unknown factor (e. g. a protein), which triggers a response as soon as it has reached a threshold concentration (Lees 1973, see also Colizzi et al. this issue). The ‘hourglass clock’ can be completely independent of the circadian clock, but it can also be a heavily damped circadian clock that loses its ability to oscillate in constant darkness (Saunders and Lewis 1987). The circadian clock of *D. ezoana* is such a heavily damped clock, which stops to oscillate within very few days after transfer into constant darkness (Vaze and Helfrich-Förster 2016; Menegazzi et al. 2017; Bertolini et al. 2019), but we do not know yet whether it is involved in night/day length measurement for diapause induction.

Two main principles of photoperiodic time measurement involving the circadian clock are discussed: ‘external coincidence’ and ‘internal coincidence’ (Saunders 1978 and this issue). In the external coincidence model, the clock is supposed to consist of a single oscillator (or group of oscillators), in which long-night induction of diapause is brought about, when a particular light-sensitive or photoinducible phase of the clock falls in the dark (Pittendrigh and Minis 1964; Pittendrigh 1966). In internal coincidence, the clock is thought to comprise two circadian oscillators (or groups of oscillators), one phase set by the dawn transition and one by dusk, whose mutual phase relationship thus changes as the photoperiod either shortens or lengthens (Pittendrigh 1960, 1972). Day length is encoded in the distance between the two oscillators. The fundamental difference between the two models, therefore, is that in internal coincidence light has only a single role (entrainment of the constituent oscillations), whereas in external coincidence it has two (entrainment and induction) (Saunders 1978). For the parasitic jewel wasp, *Nasonia vitripennis*, the internal coincidence appears to be valid, while for its host, the flesh fly, *Sarcophaga argyrostoma*, rather the external coincidence for measuring night length seems appropriate (Saunders 1978). The fruit fly, *Drosophila melanogaster*, has been proposed to be a good model for internal coincidence because it possesses morning and evening clocks that had even been anatomically defined (Helfrich-Förster 2009; Yoshii et al. 2012). However, the morning and evening clocks may mainly be important for adapting fly activity rhythms to different day lengths and not for measuring day length for induction of reproductive dormancy. This has two main reasons: (1) *D. melanogaster* flies have only a shallow dormancy that depends not only on day length but also strongly on temperature (see above), (2) *D. melanogaster period* mutants with slow or fast clocks or without functional clock still showed photoperiodic responses that resembled wild-type flies suggesting that the circadian clock is not involved in day length measuring (Saunders 1990).

*Period* is the first core clock discovered gene and codes for the PERIOD protein (PER) (Konopka and Benzer 1971). In a first negative feedback loop, PER forms dimers with *Drosophila* TIMELESS (d-TIM), the second discovered clock protein (Sehgal et al. 1994), and together PER and d-TIM block their own transcription by inhibiting their transcriptional activators CLOCK (CLK) and CYCLE (CYC) (reviewed in Hardin 2011). In a second loop the clock proteins VRILLE (VRI) and the PAR Domain Protein 1ε (PDP1ε) lead to a rhythmic transcription of the *clock* gene. Another important component of the clock is the light sensitive CRYPTOCHROME (also called Drosophila CRY, or d-CRY) (Emery et al. 1998; Stanewsky et al. 1998), which is expressed in about half of the clock neurons of all *Drosophila* species investigated so far (Yoshii et al. 2008; Hermann et al. 2013). D-CRY has an important role in transmitting light-information directly to the molecular clock network by leading to the degradation of TIM (Ceriani et al. 1999). *per*^*0*^ mutants lack the PER protein and show arrhythmic behaviour under constant conditions. But they still possess d-TIM and d-CRY meaning that light-activated d-CRY can degrade d-TIM every morning. Indeed, under light–dark cycles, *per*^*0*^ mutants appear to retain residual rhythmicity, which is mainly evident in rhythmic morning activity (Helfrich and Engelmann 1987; Helfrich-Förster 2001).

Given all this evidence, fruit flies and their *period* mutants are not the perfect model to study photoperiodic effects and the role of the circadian clock in them. Nevertheless, recent studies have shown functional connections between the circadian clock neurons and the neurohormonal system of *D. melanogaster* and have demonstrated influences of the circadian clock on reproductive dormancy strongly suggesting that the circadian clock is involved in photoperiodic responses (Nagy et al. 2019; Abrieux et al. 2020; Hidalgo et al. 2023; Kurogi et al. 2023).

Here, we investigated the rhythmic locomotor activity of the strongly photoperiodic *D. ezoana* flies under light–dark cycles with different Zeitgeber periods and photoperiods to clarify whether their circadian clocks are suited to measure night/daylength for diapause induction. Our first aim was to characterize the *D. ezoana* clock in detail, we wanted to know its strength and see whether it is an hourglass or a weak self-sustained circadian oscillator. While an hour-glass clock will keep the same phase to lights-on (or lights-off) under all Zeitgeber periods, the phase relationship of a selfsustained circadian oscillator to lights-on will change with photoperiod and Zeitgeber period. Under Zeitgeber cycles with short period (e.g. 18 h) it will have later phases while under Zeitgeber cycles with long period (e.g. 30 h) it will have earlier phases (Aschoff and Wever 1962; Hoffmann 1969; Aschoff and Pohl 1978). The stronger the circadian clock, the larger are the expected phase changes, while weak damped clock are expected to behave almost like hour-glass clocks and keep a similar phase to lights-on (lights-off) under all Zeitgeber cycles (Roenneberg et al. 2003, 2005).

We previously tested the effects of Zeitgeber cycles with periods between 18 and 30 h on the critical day/night length for diapause induction in female *D. ezoana* flies and found that *D. ezoana* requires a minimum night length of ~ 7 h for diapause induction, irrespective of the Zeitgeber period (Vaze and Helfrich-Förster 2016). We concluded that the flies use a ‘hourglass’ clock or highly damped-oscillator-based clock for measuring night length. In the present study, we applied the same Zeitgeber cycles at the same environmental temperature (17 °C) and measured locomotor activity rhythms and their phase relationship to lights-on/ lights-off to determine, whether the clock underlying these rhythms behaves like an ‘hourglass’ or circadian clock and how it may measure daylength for diapause induction.

## Materials and methods

### Fly materials

The experiments were conducted with the *D. ezoana* strain 1240 J8 (isofemale line) established from the progeny of flies collected from Oulanka, Finland (66 °N, 2 °E) in the summer of 2008 (Salminen et al. 2015). Furthermore, the laboratory strain *CantonS* of *D. melanogaster* was used for comparison. The flies have been reared at laboratory room temperature (approximately 20 °C) under constant light (approximately 150 μWcm^−2^) on standard cornmeal–yeast–agar food. Eggs were collected over 2–3 consecutive days from parent vials containing five females and five males per vial. Freshly emerged adults were collected every day and maintained at a density of approximately 25–30 flies per food vial under rearing conditions. Approximately 4-day-old female or male flies were used for the locomotor activity experiments with both species.

### Experimental design of the Zeitgeber cycle experiments

Zeitgeber cycles of 18 h, 21 h, 24 h, 27 h and 30 h periods and three different daylengths (photoperiods) were tested: 50% light/50% dark, 66.6% light/33.3% dark and 83.3% light/16.6% dark (Table 1).

**Table 1:** Duration of light and dark phases under the different photoperiods and Zeitgeber cycles

	Photoperiod 50%		Photoperiod 66.6%		Photoperiod 83.3%	
Zeitgeber period (T)	Light phase (h)	Dark phase (h)	Light phase (h)	Dark phase (h)	Light phase (h)	Dark phase (h)
*T* = 18 h	9	9	12	6	15	3
*T* = 21 h	10.5	10.5	14	7	17.5	3.5
*T* = 24 h	12	12	16	8	20	4
*T* = 27 h	13.5	13.5	18	9	22.5	4.5
*T* = 30 h	15	15	20	10	25	5

### Locomotor activity recordings

For activity recording, unmated female or male flies were collected on the first day after emergence under gaseous CO_2_ anaesthesia and maintained on standard cornmeal–yeast–agar food for 4 days. Individual flies were transferred into glass tubes (5 cm length; 7 mm diameter for *D. ezoana*, and 5 mm diameter for *D. melanogaster*) containing sugar-agar medium (4% sucrose, 2% agar in water) on day 5. About 32 flies were recorded for 8–15 days in *Drosophila* Activity Monitors (Trikinetics Inc., Waltham, Massachusetts) under one of the different light–dark schedules listed in Table 1. The light intensity was set to 150 μWcm^−2^ and the temperature was kept at 17 °C throughout the experiment to simulate the same environmental conditions we had in our previous study to determine diapause incidence (Vaze and Helfrich-Förster 2016).

### Analysis of activity data

To reveal the activity pattern of the flies, activity data were plotted as individual and average actograms using the ImageJ plug-in actogramJ (Schmid et al. 2011). Furthermore, individual and average activity profiles were calculated for each fly group as described in Schlichting and Helfrich-Förster (2015). The activity profiles shown in Fig. 3 were smoothed with a moving average filter over 51 of the 1-min values.

**Fig. 1:**
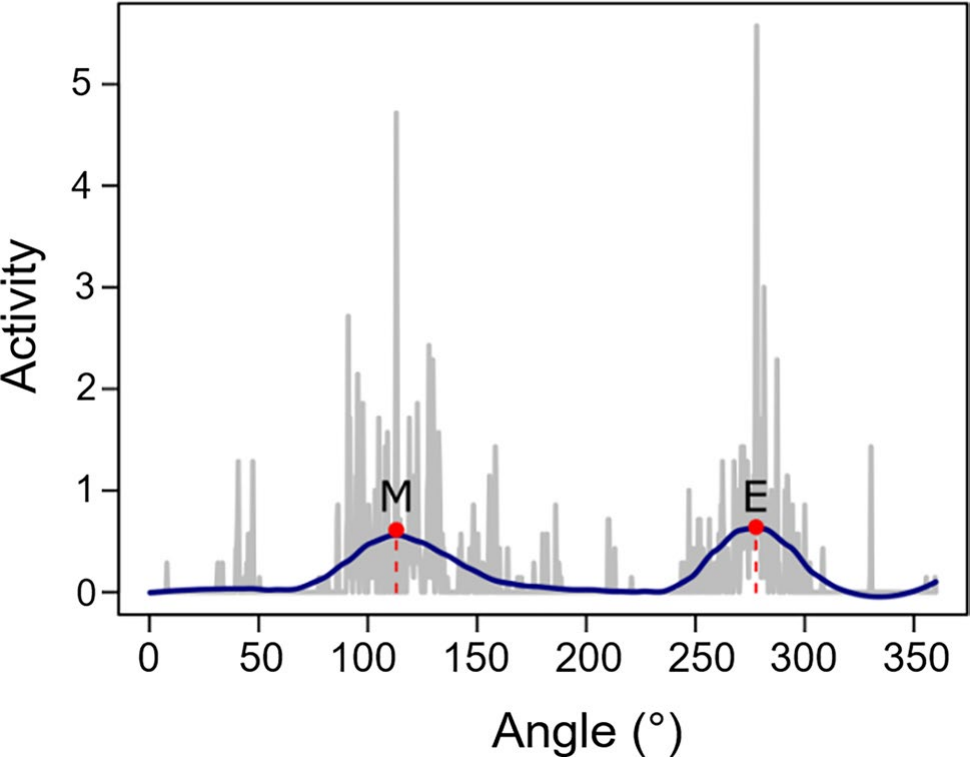
A single *D. ezoana* fly activity averaged over 5 days. The activity is shown in grey, the smoothened curve in blue, peaks found by the algorithm are shown in red, stippled lines are showing the angular value of morning (M) and evening (E) peaks

**Fig. 2:**
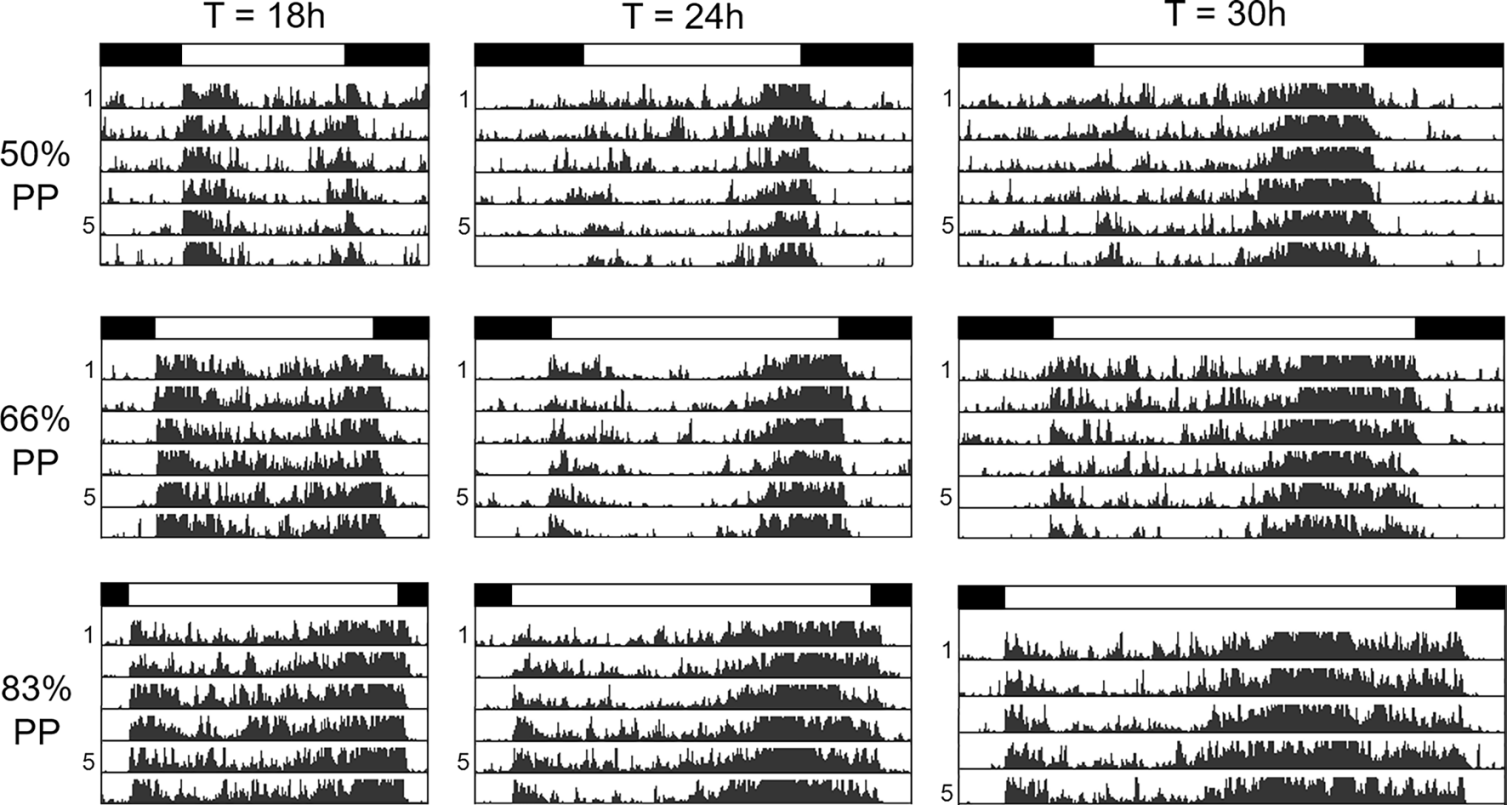
Average actograms of 32 *D. ezoana* female flies, respectively, recorded under short, normal and long Zeitgeber periods (*T* = 18 h, *T* = 24 h and *T* = 30 h) at three different photoperiods (PP50%, PP66.6%, and PP83.3%). Black and white bars on top indicate the light–dark cycles

**Fig. 3:**
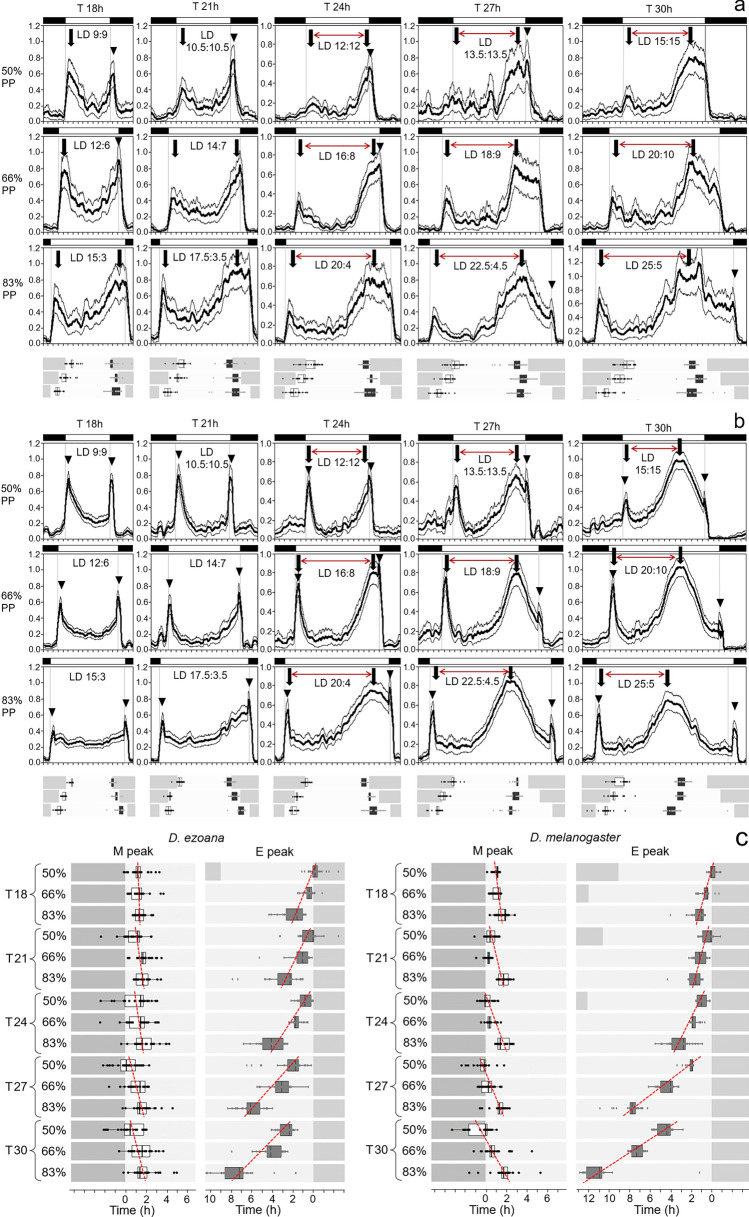
Average activity profiles of *D. ezoana* females (**a**) and *D. melanogaster* males (**b**) at the three photoperiods (PP) and five Zeitgeber periods (T). Thick arrows mark the estimated peaks of morning and evening activity, and red horizontal arrows indicate the distance between them, while arrowheads mark lights-on and lights-off startle responses. The latter were mainly present in *D. melanogaster* males. Particularly at shorter Zeitgeber periods, the startle responses overlapped with the endogenous morning and evening activity peaks on the average activity profiles, which made phase determination by eye difficult. Nevertheless, the peaks could be unequivocally determined in most individual flies by our algorithm in R, and these are indicated in box plots below the average activity profiles (morning peaks: white bars; evening peaks: black bars). **c** Phases of morning (M) and evening (E) peaks of *D. ezoana* in hours after lights-on and before lights-off, respectively. Red broken lines indicate the slopes of peak changes with photoperiod (50%, 66.6% and 83.3%). T: Zeitgeber period

The maxima of morning and evening activity bouts were calculated on the daily activity averaged over 5 days for every single fly. To fit our activity data, we used a modified code for R (v4.1.3, R Development Core Team (2018): https://www.r-project.org/) originally from William A. Huber (https://stats.stackexchange.com/q/36326). Briefly, the data were duplicated to get a double plot, it was then smoothened by local regression with a 0.02 span. A second curve was created by resampling the smoothed data via moving average filter over 11 data points. The peaks are then calculated as the intersection points between the curve generated by moving average and the original activity data (Fig. 1 show the calculated peaks in a single plot of a single fly). A peak was considered as morning if falling in the first half of the light phase and as evening if falling in the second half. Additionally, to discriminate between two close peaks, the one with the highest activity value was chosen. All the values were subsequently checked on single fly actograms to remove possible mistakes. The boxplots in Figs. 2 and 3 were generated using ggplot2 (Wickham 2016).

## Results

### *D. ezoana* flies entrain to all Zeitgeber cycles and show morning and evening activity

As shown previously for *D. melanogaster* flies (Helfrich and Engelmann 1987; Helfrich-Förster 2001; Vaze and Helfrich-Förster 2021), the *D. ezoana* females entrained well to all Zeitgeber periods (T) and photoperiods (PP), even to the extremes (*T* = 18 h, PP50% and *T* = 30 h, PP83.3%) (Fig. 2). To our surprise, they showed not only the evening activity bouts as previously described under 24 h cycles (Menegazzi et al. 2017) but also morning activity bouts that were separated by a shallow siesta from evening activity. Morning activity was most prominent at short Zeitgeber periods (*T* = 18 h and *T* = 21 h) but still present at the longest Zeitgeber period and photoperiod (*T* = 30 h and 83.3% PP) (Figs. 2 and 3a, arrows). In contrast to *D. melanogaster* males (Fig. 3b), that showed the well-described prominent startle responses at lights-on and lights-off, *D. ezoana* females virtually lacked such startle responses. Only at lights-off, moderate startle responses were sometimes visible (Fig. 3a. arrowheads). To test whether the startle responses are sex-specific and only found in male flies, we also recorded *D. ezoana* males under *T* = 21 h and *T* = 27 h at the three different photoperiods (Fig. S1). We did not detect evident startle responses. Furthermore, the morning activity and the siesta were also clearly visible in male *D. ezoana* flies. We conclude that the activity of male and female *D. ezoana* flies is similar and that the differences observed between the present study and the previous study may be due to the different conditions used. Here we used only unmated males and recorded them at 17 °C under rectangular light–dark cycles, whereas in the previous study, we used mated males and recorded them at 20 °C under simulated dawn and dusk (Menegazzi et al. 2017).

### Increasing photoperiod strongly advances the phase of morning activity and moderately delays that of evening activity

**Fig. 4:**
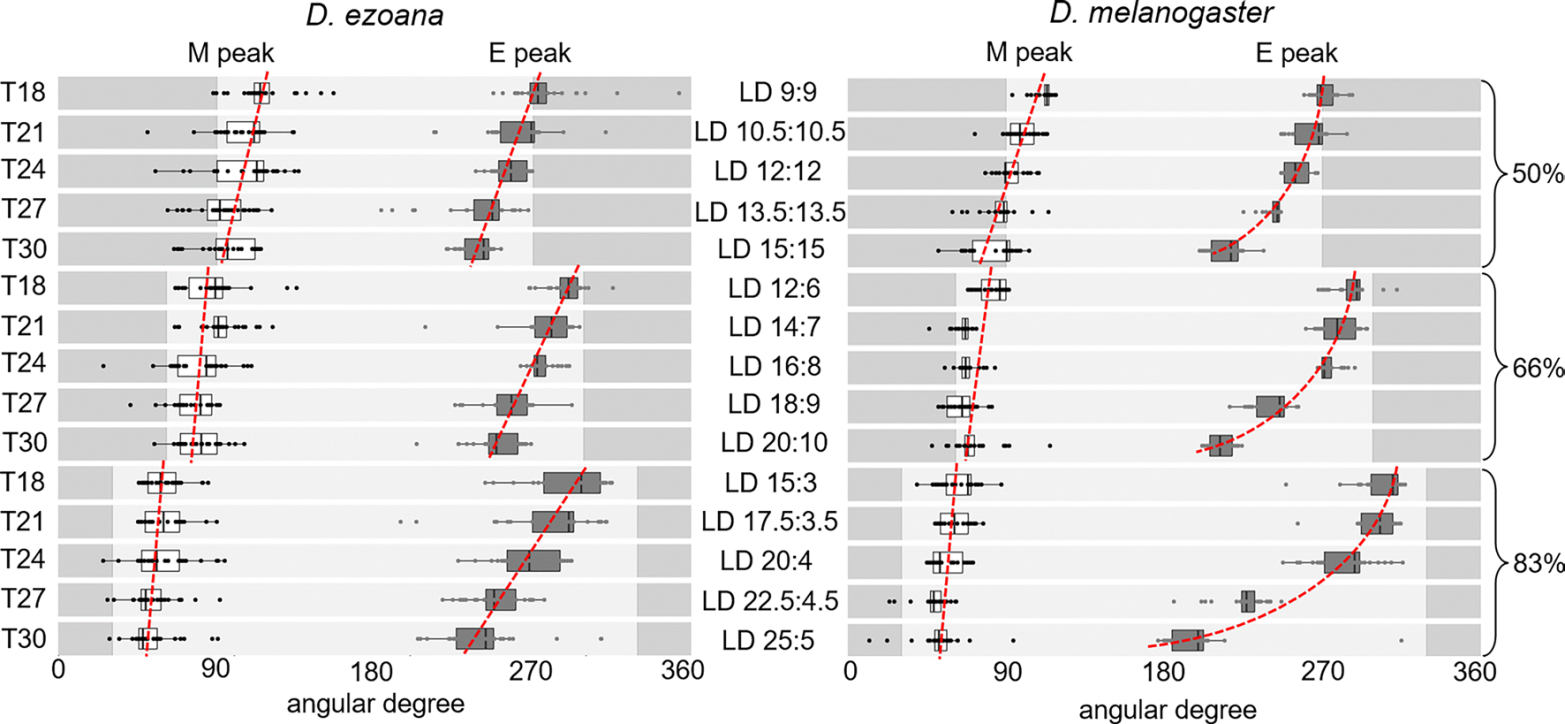
Phases of morning (M) and evening (E) activity peaks in angular degrees in dependence of the Zeitgeber period (T). The red broken lines are eye-fitted slopes demonstrating the phase advances of M and E peaks under increasing Zeitgeber periods. The graphs are shown under all three photoperiods (50%, 66.6% and 83.3%)

In both species, morning activity tracked dawn and evening activity tended to track dusk under increasing photoperiod. Consequently, the phase relationship between morning and evening activity increased with increasing photoperiod (Fig. 3; horizontal red arrows at *T* = 24 h, 27 h and 30 h). These changes can be interpreted as seasonal adaptations and are well investigated at the natural Zeitgeber period of 24 h in different fly species (Menegazzi et al. 2017; Beauchamp et al. 2018; Bertolini et al. 2019). While morning activity was close to dawn at all three photoperiods, evening activity had a limited capability to couple to dusk and occurred clearly before dusk at very long photoperiods (Fig. 3). The ability of evening activity to track dusk at very long photoperiods has been found to correlate with the latitude the flies stem from (Rieger et al. 2012; Beauchamp et al. 2018; Deppisch et al. 2022). It was particularly pronounced in flies from high latitudes and thought to be an adaptation to the very long days of summer. In the present study, we did not see a difference in the phase of evening activity between the two species, at the Zeitgeber period of 24 h. However, at longer Zeitgeber periods (*T* = 27 h and *T* = 30 h), the high-latitude *D. ezoana* flies were able to significantly increase the phase relationship between morning and evening activity compared to *D. melanogaster* flies (Figs. 3, 4). As can be seen in Fig. 3 and verified by a two-way ANOVA (Table 2), the ability of evening activity to track dusk depended strongly on the photoperiod. Its distance to lights-off significantly increased with increasing photoperiod (Fig. 3c). This means that the evening oscillator of *D. ezoana* is a rather strong oscillator that cannot easily shift towards light-off. The evening oscillator of *D. melanogaster* appears even stronger because its distance to lights-off was larger under long photoperiods than that of *D. ezoana* (Fig. 3c). In other words, its peak phase in relation to lights-off depended, to a higher degree, on photoperiod (Table 2).

**Table 2:** Dependence of morning and evening activity peak phase (to lights-on and lights-off, respectively) on photoperiod (PP) and Zeitgeber period (T) in *D. ezoana* females and *D. melanogaster* males as revealed by a two-way ANOVA

Dependence	*Drosophila ezoana*				*Drosophila melanogaster*			
	Morning peak		Evening peak		Morning peak		Evening peak	
	*F* ratio^a^	Signifi-cance	*F* ratio^a^	Signifi-cance	*F* ratio^a^	Signifi-cance	*F* ratio^a^	Signifi-cance
PP	*F*_(2,454)_ = 30.66	*p* < 0.001	*F*_(2,454)_ = 341.38	*p* < 0.001	*F*_(2,454)_ = 165.89	*p* < 0.001	*F*_(2,454)_ = 867.88	*p* < 0.001
*T*	*F*_(4,454)_ = 3.43	*p* = 0.009	*F*_(4,454)_ = 192.87	*p* < 0.001	*F*_(4,454)_ = 16.59	*p* < 0.001	*F*_(4,454)_ = 1607.19	*p* < 0.001
Interaction between PP and *T*^b^	*F*_(8,454)_ = 3.16	*p* = 0.002	*F*_(8,454)_ = 7.13	*p* < 0.001	*F*_(8,454)_ = 9.11	*p* < 0.001	*F*_(8,454)_ = 103.52	*p* < 0.001

^a^The higher the *F* ration, the higher the dependence of peak phase on PP or T

^b^A significant interaction between PP and T means that the dependence of morning or evening activity on PP was different at different Ts

In our previous studies, we did not determine the peak phases of morning activity, because it was difficult to distinguish the morning peak from the lights-on peak, which is particularly pronounced in *D. melanogaster*. The lack of the lights-on peak in *D. ezoana* encouraged us to determine also the peak phase of morning activity with our new algorithm in R (Fig. 1). We found that the phase of the morning peak depended on day length (Table 2) occurring later in respect to lights-on under long photoperiods (Fig. 3c). This confirms its nature as endogenous morning oscillator. However, the morning oscillator appears to be a weak oscillator since it can be easily shifted and therefore remains rather close to lights-on. In *D. melanogaster*, the morning peak depended even stronger on photoperiod than in *D. ezoana* (Table 2) suggesting that *D. melanogaster* has not only a stronger evening oscillator but also a stronger morning oscillator than *D. ezoana* (Fig. 3c). This result also shows us that our algorithm determining peak phases is not significantly affected by the lights-on peak.

### Peak analysis under the different Zeitgeber periods confirms our conclusion that the evening oscillator is stronger than the morning oscillator

The phase relationship of circadian oscillators to lights-on and lights-off is also expected to change with Zeitgeber period, and most likely in a more dramatic manner than with photoperiod. Under Zeitgeber cycles with short period (e.g. *T* = 18 h) the oscillation is expected to phase lag lights-on/lights-off, while it is expected to phase lead both under Zeitgeber cycles with long period (e.g. *T* = 30 h). The stronger the circadian clock, the larger are the expected phase changes relative to lights-on and lights-off (the steeper the slope of T-phase relationship), while weak damped clock are expected to behave almost like hour-glass clocks and keep a similar phase to lights-on/lights-off under all Zeitgeber periods (the shallower the slope). To visualize the relation between morning and evening peaks to lights-on and lights-off under different Zeitgeber periods, we plotted the daily cycles not in real hours but in phase angles (degrees). The entire cycle equals an angular degree of 360° with lights-on at 90 °C and lights-off at 270° under a photoperiod with equal length of light and darkness (50% PP). Under 66.6% photoperiods, lights-on is at 61° and lights-off at 299°, while under 83.3% photoperiod they are at 31° and 329°, respectively (Fig. 4). We found that in both *Drosophila* species morning and evening activity peaks behaved like it is expected for circadian oscillators: The morning peak became slightly earlier with increasing Zeitgeber period, and this was most evident under photoperiods with equal length of day and night (50% PP) and again more pronounced in *D. melanogaster* flies than in *D. ezoana* flies (Fig. 4). The evening peaks became clearly earlier with increasing Zeitgeber period, and this was most pronounced under long photoperiods and particularly strong in *D. melanogaster*. While in *D. ezoana* the evening peak advanced linearly with increasing Zeitgeber period, its advance curve was parabolic in *D. melanogaster* (Fig. 4). Most likely the evening oscillator was at its entrainment limit in *D. melanogaster* at the 30 h Zeitgeber period, while it could still stably entrain in *D. ezoana*. These results confirm our conclusion that the evening oscillator is stronger than the morning oscillator in both species and that it is especially strong in *D. melanogaster*.

### *D. ezoana* females appear to measure the time passing from lights-off until the morning activity peak for determining diapause onset

**Fig. 5:**
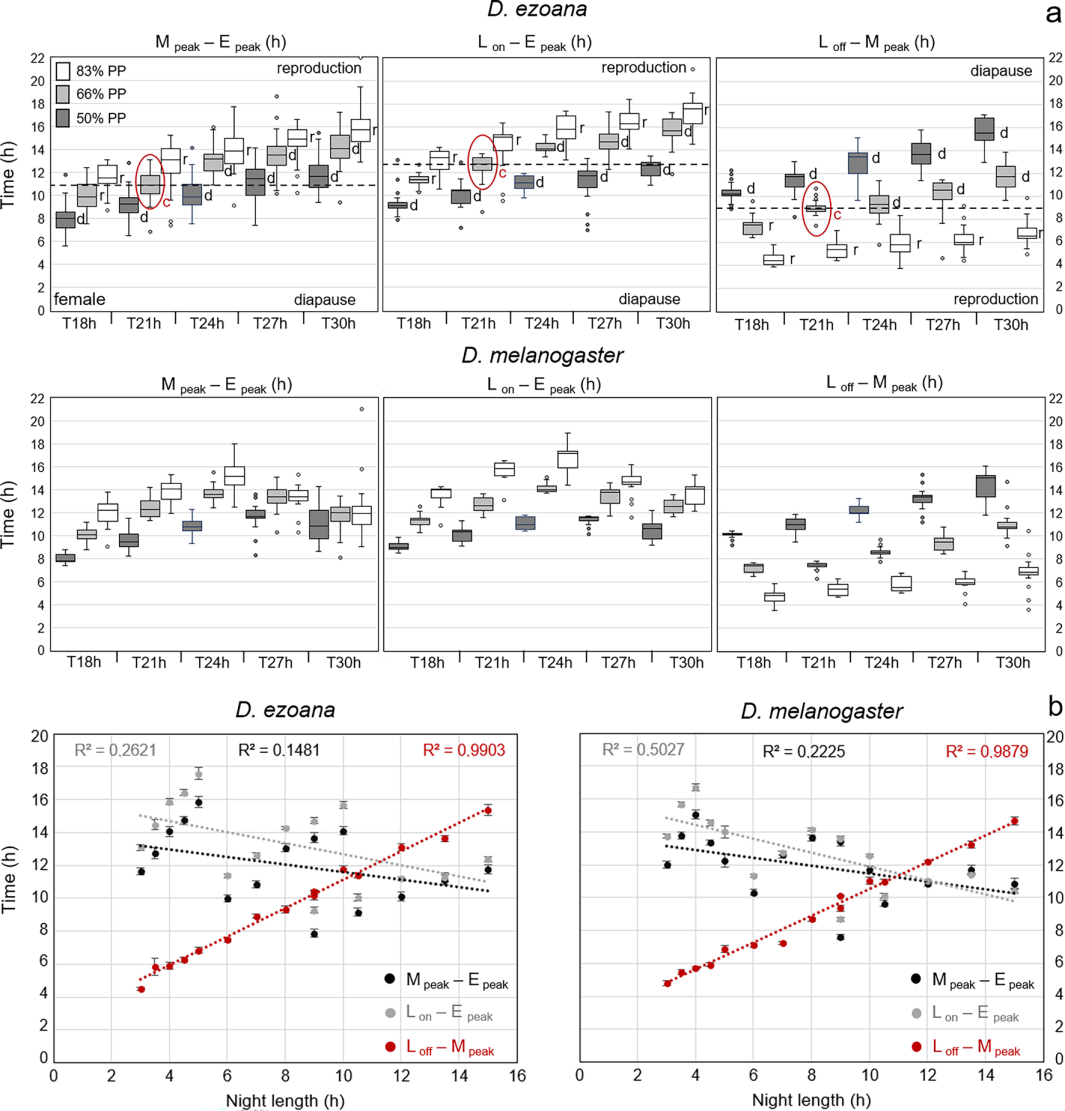
Possible mechanisms for measuring night length for diapause induction in *D. ezoana*. **a** Boxplots showing the time passed between morning and evening peaks (*M*_peak_ – *E*_peak_), lights-on and evening peak (*L*_on_ – *E*_peak_) and light-off and morning peak (*L*_off_ – *M*_peak_) for all flies under the different Zeitgeber periods (T) and photoperiods (PP). The horizontal stippled line represents the critical night length (c) for diapause induction as determined by Vaze and Helfrich-Förster (2016). All boxplots above or below this line should either result in diapause (d) or in reproduction (r). The values for *D. melanogaster* are shown for comparison. **b** Correlation between *M*_peak_ – *E*_peak_, *L*_on_ – *E*_peak_, and *L*_off_ – *M*_peak_ with night length for *D. ezoana* and *D. melanogaster*. R^2^: correlation coefficients

So far, we showed that the activity rhythm of *D. ezoana* is controlled by morning and evening oscillators. Both oscillators fulfil the criteria for endogenous oscillators, whereby the evening oscillator is considerably stronger than the morning oscillator. With increasing day length both oscillators tended to track dawn and dusk, respectively, but the stronger evening oscillator could do so less well. Nevertheless, the phase relationship between morning and evening activity steadily increased with increasing photoperiod and could theoretically be used by the flies to measure day length (internal coincidence). Alternatively, the flies could measure the time passing between lights-on and the peak of the evening oscillator, or the time passing between lights-off and the peak of the morning oscillator. Both alternative possibilities are more in line with the external coincidence model. Since we have measured diapause under different Zeitgeber periods and photoperiods in our previous study (Vaze and Helfrich-Förster 2016), we had now the possibility to test which of the mentioned possibilities fits best to the data. For this purpose, we plotted for all flies the time (in h) that passed between the peaks of morning and evening activity (*M*_peak_ – *E*_peak_) for all Zeitgeber cycles and photoperiods (Fig. 5a). We did the same for the time that passed between lights-on and the evening peak (*L*_on_ – *E*_peak_) and for the time that passed between lights-off and the morning peak (*L*_off_ – *M*_peak_) (Fig. 5a). Our previous study had revealed that *D. ezoana* measures absolute night length for entering diapause and requires a minimum night length of ~ 7 h. Among all the light regimes in our study, this night length is reached under a Zeitgeber period of 21 h and 66.6% photoperiod (red circled boxplots marked by “c” and horizontal broken lines in Fig. 5a). The night lengths in other light regimes were either shorter than ~ 7 h and should lead to reproduction (marked by “r” in Fig. 5a) or longer than ~ 7 h and should lead to diapause (marked by “d” in Fig. 5a).

Interestingly, among the times passed between *M*_peak_ and *E*_peak_, *L*_on_ and *E*_peak_, and *L*_off_ and *M*_peak_, the *L*_off_ – *M*_peak_ distance showed a strong positive correlation (R^2^ = 0.99) with night length, whereas the distances between *M*_peak_ – *E*_peak_ and *L*_on_ – *E*_peak_ were negatively correlated with night length (Fig. 5b). The duration of *L*_off_ – *M*_peak_ (~ 9 h) is closest to ~ 7 h night length required by *D. ezoana* to enter diapause under a Zeitgeber period of 21 h and 66.6% photoperiod. Furthermore, all *L*_off_ – *M*_peak_ values longer than ~ 9 h led to diapause in our previous study (Vaze and Helfrich-Förster 2016), while all shorter values led to reproduction (Fig. 5a). This suggests that *D. ezoana* flies most likely measure night length to induce diapause by tracking the time passed between lights-off and an early phase of the morning oscillator.

The values for *D. melanogaster* are just shown for comparison because these flies have no clear photoperiodically controlled diapause. Nevertheless, the distribution of the boxplots makes it quite unlikely that *D. melanogaster* could use the time passing between morning and evening peaks as reliable daylength measurement. The same appears true for the time passing between lights-on and the evening peak. As in *D. ezoana*, the strongest correlation was found between night length and the time passed between *L*_off_ and *M*_peak_ in *D. melanogaster* (Fig. 5b).

## Discussion

Our data demonstrate that the activity rhythm of *D. ezoana* and *D. melanogaster* is controlled by morning- and evening oscillators with different properties. As shown previously, light shortens the period of the morning oscillator and lengthens that of the evening oscillator (Rieger et al. 2006) enabling the two to track dawn and dusk, respectively, when daylength increases. Here, we show that morning and evening oscillators not only differ in their responses to light but also in their strength: the morning oscillator is much weaker than the evening oscillator evident from the stronger latching of morning oscillator to dawn than that of the evening oscillator to dusk across increasing photoperiod (Fig. 3B, C). Although weaker, the morning oscillator does not appear to behave like an ‘hourglass’. The phase relationship of morning oscillator shows systematic photoperiod and Zeitgeber period dependence (Fig. 4) as expected of the circadian oscillator suggesting that it is a true but damped circadian oscillator (Aschoff and Wever 1962; Hoffman 1969; Aschoff and Pohl 1978).

Although *D. ezoana* flies have morning and evening oscillators that follow dawn and dusk more closely than those of *D. melanogaster*, they do not appear to use internal coincidence to measure photoperiod for diapause initiation. The external coincidence model assumes a circadian rhythm of a photoinducible substance “c” that accumulates above a certain threshold during long diapause-inducing nights. During short nights, the peak phase of substance “c” is exposed to light, which leads to its degradation, which in turn results in the absence of diapause (Pittendrigh and Minis 1964). We previously showed that the external coincidence involving a damped circadian oscillator may underlie the absolute night length measurement by *D. ezoana* (Vaze and Helfrich-Förster 2016). Our analysis of morning peak phases across different light regimes shows that the time passed between *L*_off_ and *M*_peak_ strongly correlates with night length and the diapause phenotype (Fig. 5). Taken together, then, these results suggest that *D. ezoana* measures the length of the night by tracking the time between lights-off and the light-sensitive phase of the morning oscillator by external coincidence.

### Comparison with other insects

A series of clever experiments with night-interrupting light-pulses (Nanda and Hamner 1958; Bünsow 1960) have been performed in aphids, flies and pitcher-plant mosquitos to test the role of the circadian clock in photoperiodic time measurement (Lees 1973; Saunders this issue; Bradshaw et al. this issue). These indicated that flesh and blow flies rely on the circadian clock (external coincidence) for photoperiodic time measurement (Saunders 2021), aphids use an interval timer (‘hourglass’; Lees 1973), and the response of pitcher-plant mosquitos depends on their geographical origin (Bradshaw et al. this issue). The pitcher-plant mosquito, *Wyeomyia smithii*, belongs to one of the rare species, in which the photoperiodic response has been systematically investigated in populations living at different latitudes altitudes (Bradshaw et al. this issue). These studies showed that critical photoperiod has increased while the apparent involvement of the circadian clock has decreased with latitude, meaning that external coincidence is used at low latitudes and an ‘hourglass’ at high latitudes. We wonder if the apparent lesser influence of the circadian clock could be due to the fact that it becomes weaker (more damped) with increasing latitude. Lower amplitudes of overt circadian rhythms at high latitudes have been reported for several species (Lankinen 1993; Kyriacou et al. 2008; Hut et al. 2013; Helfrich-Förster et al. 2018; Abe et al. 2021). As mentioned earlier, high-latitude drosophilids have a dampened circadian clock and become rapidly arhythmic under constant conditions (Yoshida and Kimura 1995; Kauranen et al. 2012; Vaze and Helfrich-Förster 2016; Lankinen et al. 2021; Bertolini et al. 2019). The same is true for aphids (Beer et al. 2017; Matsuda 2023). This suggests that the negative Nanda-Hamner and Bünsow responses gained in some of these species may be due to the weaker underlying clocks.

Nevertheless, we show here that external coincidence is most likely also used by *D. ezoana* suggesting that it may be a general mechanism of photoperiodic time measurement in flies, mosquitos, and aphids. It may be even independent of the type of photoperiodic response. While *Calliphora*, *Lucilia*, *Chymomyza* and *Wyeomyia* species diapause as larvae (Enomoto 1982; Saunders 2021; Bradshaw et al. this issue), *Sarcophaga* diapauses as pupa (Denlinger 1972), *Protophormia* and *Drosophila* species diapause as adults (Lumme and Lakovaara 1983; Numata and Shiga 1995), and aphids show a fascinating annual life cycle in which parthenogenetic and sexual reproduction alternate (Colizzi et al. this issue).

There is another unifying feature in flies, mosquitos and aphids: they all possess d-TIM and light-sensitive d-CRY (Kotwica-Rolinska et al. 2022; Deppisch et al. 2023). d-CRY interacts with d-TIM and provokes its degradation upon exposure to light (Ceriani et al. 1999; see below). In contrast, hymenopterans to which *Nasonia vitripennis* belongs, lack d-CRY and d-TIM and possess only light-insensitive mammalian CRY (also called m-CRY) that interacts with PER (Kotwica-Rolinska et al. 2022; Deppisch et al. 2023). Thus, hymenopterans lack the immediate light response elicited by the degradation of d-TIM by d-CRY, and fittingly, they use internal coincidence to measure daylength (Saunders 2021).

### Putative molecular correlates for night length measurement in flies

Evidence from multiple sources suggest the role for clock protein TIMELESS in the regulation of insect photoperiodic diapause. In *D. melanogaster*, a latitudinal cline in diapause incidence has been associated with a *d-tim* polymorphism (Sandrelli et al. 2007; Tauber et al. 2007). The recently evolved long form of-d-TIM is less light-sensitive than the ancient short form of d-TIM (Sandrelli et al. 2007; Deppisch et al. 2022; Lamaze et al. 2022), could therefore persist at higher levels during the day and accumulate faster to diapause inducing threshold levels in the night, which will then result in a dormancy at shorter nights (longer days). In other words, the critical day length will be shifted to longer days, and exactly this was observed (Tauber et al. 2007; Pegoraro et al. 2017). Latitudinal differences in the expression of d-TIM were also observed in *D. triauraria* (Yamada and Yamamoto 2011) and *Wyeomyia smithii* (Mathias et al. 2005). Consistent with these observations, mutations in the *tim* gene affect photoperiodic responses. *d-tim*^*0*^ mutants of *D. melanogaster* did not reduce the size of their ovaries under short photoperiods and the overexpression of *d-tim* resulted in a reduction of ovary size, which indicates the beginning of dormancy (Abrieux et al. 2020). *d-tim*-mutants of the drosophilid fly *Chymomyza costata* show a dramatical suppression of *d-tim* transcription in the brain and lose their photoperiodic responses (Stehlík et al. 2008; Kostál and Shimada 2001; Kobelková et al. 2010).

Flies, which belong to the cyclorrhaphan Diptera, are unique in that they are the only insects in which PER has only d-TIM as an interaction partner, since the light-insensitive m-CRY has been lost (mosquitos and aphids still possess m-CRY) (Kotwica-Rolinska et al. 2022; Deppisch et al. 2023). Every cycle the *tim* transcription restarts as soon as TIM and PER have disappeared and reaches its maximum at the beginning of the night so that *tim* translation can immediately start at lights-off. TIM accumulates over the night and peaks during its end before it is degraded in the morning through interaction with light-activated d-CRY. Thus, in theory d-TIM dynamics can potentially work like an hour-glass timer and measure night-length to induce diapause (Bradshaw and Holzapfel 2007). This may explain why *per*^*0*^, *per*^*s*^ and *per*^*l*^ mutants have largely unaltered photoperiodic responses (Saunders 1990).

Although all these studies suggest a strong causal relationship of the *d-tim* locus with diapause, it is unclear whether this is mediated by its clock function. It might also be caused by pleiotropic effects of TIM on photoperiodism (Bradshaw and Holzapfel 2007). However, if circadian involvement is real, d-TIM might provide the missing link between the circadian clock and the photoperiodic system at least in those insects that possess d-TIM and light-sensitive d-CRY. In the latter d-TIM could represent the rhythm of the photosensitive substance ‘c’ predicted by the external coincidence model.

### Putative anatomical correlates for night length measurement

But what is the relation between d-TIM and the morning oscillator?

**Fig. 6:**
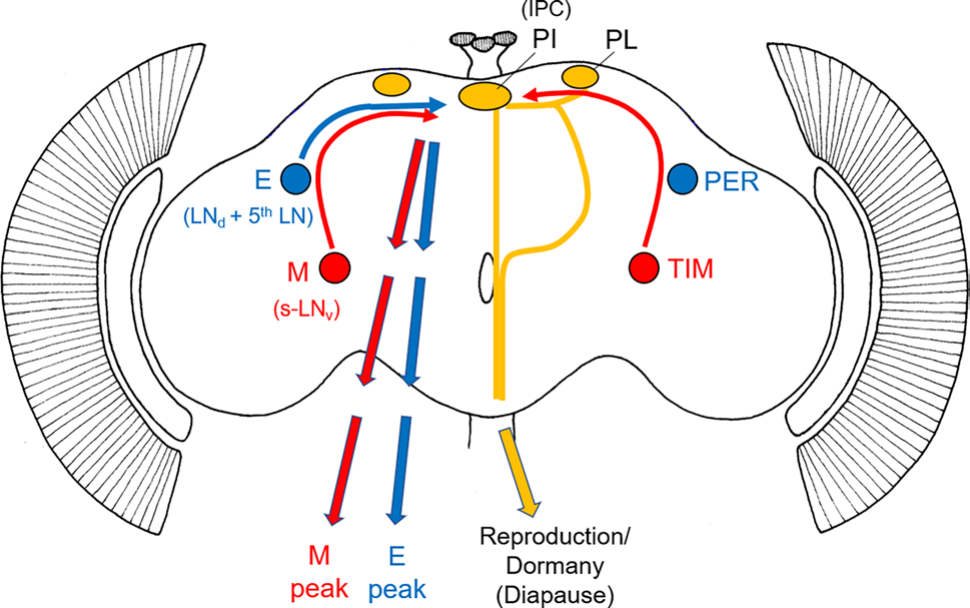
Simplified hypothetical model of how the clock neurons in the brain of fruit flies may control the rhythms of daily activity (left brain hemisphere) and mediate night length to the photoperiodic control centres in the superior brain (right brain hemisphere). M: morning neurons (= s-LN_v_, small ventrolateral neurons), E: evening neurons (= LN_d_ + 5^th^ LN, dorsolateral neurons plus 5th lateral neuron), *PER* Period, *TIM* Timeless. PI, *pars intercerebralis* with insulin-producing cells (IPC) and *pars lateralis* (PL) with other neurosecretory cells. Note that the model is mainly derived from results gained in *D. melanogaster.* The connections from the M neurons to the IPC cells have not been proven for *D. ezoana*

In *D. melanogaster*, the morning oscillator ticks in the small ventrolateral clock neurons, called s-LN_v_s while the evening oscillator ticks in the dorsolateral clock neurons, called LN_d_, or more precisely in the LN_d_ and the 5th LN (Fig. 6) (reviewed in Yoshii et al. 2012). Antibody labelling against the clock proteins PER and d-TIM at different daylengths showed that the two clock proteins are differentially regulated (Shafer et al. 2004; Vanin et al. 2012; Menegazzi et al. 2013). Under 12:12 h light dark cycles, they peak both at the end of the night. While TIM is immediately degraded after lights-on, PER starts to decrease several hours after lights-on. Under long days, the d-TIM peak remains in the night, while the PER peak is delayed, and PER remains present until the middle of the day (Menegazzi et al. 2013). In the same study, it was also found that morning activity in *D. melanogaster* always starts when d-TIM decreases, while the start of evening activity correlates with the decrease of PER, which occurs considerably later, particularly under long days (Menegazzi et al. 2013). Thus, d-TIM might be a crucial component of the morning activity, while PER appears more important for the evening activity.

Here, we hypothesize that TIM in the morning neurons controls the timing of morning activity and is involved in time measurement for photoperiodic responses, while PER in the evening neurons controls the timing of evening activity (summarized in Fig. 6). This does not mean that morning activity is independent of PER and evening activity independent of TIM. Since both clock proteins strongly interact and the entire molecular circadian cycle depends on this interaction, a clock lacking one of the two proteins is not functional. Nevertheless, morning activity can persist to some degree without PER (Helfrich-Förster 2001). In contrast, evening activity cannot persist without TIM, because TIM is needed for PER accumulation.

In *D. melanogaster*, there is already evidence that the morning neurons control not only morning activity but are also involved in the timing of dormancy. They signal directly to the insulin-producing neurosecretory cells in the *pars intercerebralis* and affect their activity (Ojima et al. 2018; Nagy et al. 2019; Hidalgo et al. 2023). The insulin-producing cells are key regulators of reproduction and diapause in numerous species (Kimura et al. 1997; Sim and Denlinger 2008, 2013; Kubrak et al. 2014).

### Open questions

In *D. ezoana*, the time-course of d-TIM and PER has not yet been analysed because the antibodies raised against *D. melanogaster* d-TIM and PER do not work reliably. However, morning and evening neurons are present as assessed with antibodies with PDP1 and d-CRY (Hermann et al. 2013). It is likely that PER and d-TIM have similar functions in *D. ezoana* as in *D. melanogaster*, and future studies must show if the two clock proteins cycle similarly out of phase under long days. Furthermore, the mechanisms by which the clock neurons signal to the neurohormonal system are still unknown in *D. ezoana*.

Without doubt there are still many open questions. Nevertheless, we show here for the first time that high-latitude *D. ezoana* flies possess morning and evening oscillators with different properties that help the flies to adapt their activity rhythms to long photoperiods. In addition, our analysis strongly suggests that the highly dampened morning oscillators measure night length for diapause induction, which will require a clear demonstration of the causal relationship between the morning oscillators and photoperiodic diapause in future studies. As discussed in the last chapters, the clock protein d-TIM might be the molecular correlate for measuring night length and the s-LN_v_s the anatomical correlates for the morning neurons. Future studies must reveal whether the here proposed hypotheses are right.

### Supplementary Information

Below is the link to the electronic supplementary material.

Supplementary file1 (DOCX 283 KB)

Supplementary file2 (TIF 270 KB)

## Data Availability

The data that support the findings of this study are available from the first author upon reasonable request.

## References

[CR1] Abe MS, Matsumura K, Yoshii T, Miyatake T (2021). Amplitude of circadian rhythms becomes weaken in the north, but there is no cline in the period of rhythm in a beetle. PLoS ONE.

[CR2] Abrieux A, Xue Y, Cai Y (2020). EYES ABSENT and TIMELESS integrate photoperiodic and temperature cues to regulate seasonal physiology in *Drosophila*. Proc Natl Acad Sci USA.

[CR3] Aschoff J, Pohl H (1978). Phase relations between a circadian rhythm and its zeitgeber within the range of entrainment. Naturwissenschaften.

[CR4] Aschoff J, Wever R (1962). On phase relationships between periods of biological rhythms and of zeitgebers. Z Vgl Physiol.

[CR5] Beauchamp M, Bertolini E, Deppisch P (2018). Closely related fruit fly species living at different latitudes diverge in their circadian clock anatomy and rhythmic behavior. J Biol Rhythms.

[CR6] Beer K, Joschinski J, Arrazola Sastre A (2017). A damping circadian clock drives weak oscillations in metabolism and locomotor activity of aphids (*Acyrthosiphon pisum*). Sci Rep.

[CR7] Bertolini E, Schubert FK, Zanini D (2019). Life at high latitudes does not require circadian behavioral rhythmicity under constant darkness. Curr Biol.

[CR8] Bradshaw WE, Fletcher MC, Holzapfel CM (this issue) Clock-talk: Have we forgotten about geographic variation. J Comp Physiol A 10.1007/s00359-023-01643-9 PMC11226528 37322375

[CR9] Bradshaw WE, Holzapfel CM (2007). Tantalizing *timeless*. Science.

[CR10] Bradshaw WE, Holzapfel CM (2010). What season is it anyway? Circadian tracking vs. photoperiodic anticipation in insects. J Biol Rhythms.

[CR11] Bünning E (1936). Die endonome Tagesrhythmik als Grundlage der photoperiodischen Reaktion. Ber Dtsch Bot Ges.

[CR12] Bünsow RC (1960). The circadian rhythm of photoperiodic responsiveness in *Kalanchoe*. Cold Spring Harb Symp Quant Biol.

[CR13] Ceriani MF, Darlington TK, Staknis D (1999). Light-dependent sequestration of TIMELESS by CRYPTOCHROME. Science.

[CR14] Colizzi FS, Martinez-Torres D, Helfrich-Förster C (this issue) The circadian and photoperiodic clock of the pea aphid. J Comp Physiol A 10.1007/s00359-023-01660-8 PMC11226554 37482577

[CR15] Denlinger DL (1972). Induction and termination of pupal diapause in *Sarcophaga* (Diptera: Sarcophagidae). Biol Bull.

[CR16] Denlinger DL (2023). Insect diapause: from a rich history to an exciting future. J Exp Biol.

[CR17] Deppisch P, Prutscher JM, Pegoraro M (2022). Adaptation of *Drosophila melanogaster* to long photoperiods of high-latitude summers is facilitated by the ls-*timeless* allele. J Biol Rhythms.

[CR18] Deppisch P, Kirsch V, Helfrich-Förster C, Senthilan PR (2023). Contribution of cryptochromes and photolyases for insect life under sunlight. J Comp Physiol A.

[CR19] Emerson KJ, Dake SJ, Bradshaw WE, Holzapfel CM (2009). Evolution of photoperiodic time measurement is independent of the circadian clock in the pitcher-plant mosquito, Wyeomyia smithii. J Comp Physiol A.

[CR20] Emery P, So W, Kaneko M (1998). CRY, a *Drosophila* clock and light-regulated cryptochrome, is a major contributor to circadian rhythm resetting and photosensitivity. Cell.

[CR21] Enomoto O (1982). Larval diapause in *Chymomyza costata* (Diptera: Drosophilidae) I. Effects of temperature and photoperiod on the development. Low Temp Sci Ser B Biol Sci.

[CR23] Goto SG (2022). Photoperiodic time measurement, photoreception, and circadian clocks in insect photoperiodism. Appl Entomol Zool.

[CR24] Hardin PE (2011). Molecular genetic analysis of circadian timekeeping in *Drosophila*. Adv Genet.

[CR25] Helfrich C, Engelmann W (1987). Evidences for circadian rhythmicity in the *per*^*0*^ mutant of *Drosophila melanogaster*. Z Naturforsch.

[CR26] Helfrich-Förster C (2001). The locomotor activity rhythm of *Drosophila melanogaster* is controlled by a dual oscillator system. J Insect Physiol.

[CR27] Helfrich-Förster C (2009). Does the morning and evening oscillator model fit better for flies or mice?. J Biol Rhythms.

[CR28] Helfrich-Förster C (2023). Biological timing: Linking the circadian clock to the season. Curr Biol.

[CR29] Helfrich-Förster C, Bertolini E, Menegazzi P (2018). Flies as models for circadian clock adaptation to environmental challenges. Eur J Neurosci.

[CR30] Hermann C, Saccon R, Senthilan PR (2013). The circadian clock network in the brain of different *Drosophila* species. J Comp Neurol.

[CR31] Hidalgo S, Anguiano M, Tabuloc CA, Chiu JC (2023). Seasonal cues act through the circadian clock and pigment-dispersing factor to control EYES ABSENT and downstream physiological changes. Curr Biol.

[CR32] Hoffmann K (1969). Zum Einfluß der Zeitgeberstärke auf die Phasenlage der synchronisierten circadianen Periodik. Z Vgl Physiol.

[CR33] Hut RA, Paolucci S, Dor R (2013). Latitudinal clines: an evolutionary view on biological rhythms. Proc Biol Sci.

[CR34] Kauranen H, Menegazzi P, Costa R (2012). Flies in the north: locomotor behavior and clock neuron organization of *Drosophila montana*. J Biol Rhythms.

[CR35] Kimura KD, Tissenbaum HA, Liu Y, Ruvkun G (1997). daf-2, an insulin receptor-like gene that regulates longevity and diapause in *Caenorhabditis elegans*. Science.

[CR36] Kobelková A, Bajgar A, Dolezel D (2010). Functional molecular analysis of a circadian clock gene timeless promoter from the Drosophilid fly *Chymomyza costata*. J Biol Rhythms.

[CR37] Konopka RJ, Benzer S (1971). Clock mutants of Drosophila melanogaster. Proc Natl Acad Sci USA.

[CR38] Kostál V (2006). Eco-physiological phases of insect diapause. J Insect Physiol.

[CR39] Kostál V, Shimada K (2001). Malfunction of circadian clock in the non-photoperiodic-diapause mutants of the drosophilid fly, *Chymomyza costata*. J Insect Physiol.

[CR40] Kotwica-Rolinska J, Chodáková L, Smýkal V (2022). Loss of *timeless* underlies an evolutionary transition within the circadian clock. Mol Biol Evol.

[CR41] Kubrak OI, Kučerová L, Theopold U, Nässel DR (2014). The sleeping beauty: how reproductive diapause affects hormone signaling, metabolism, immune response and somatic maintenance in *Drosophila melanogaster*. PLoS ONE.

[CR42] Kurogi Y, Imura E, Mizuno Y (2023). Female reproductive dormancy in *Drosophila melanogaster*is regulated by DH31-producing neurons projecting into the corpus allatum. Development.

[CR43] Kyriacou CP, Peixoto AA, Sandrelli F (2008). Clines in clock genes: fine-tuning circadian rhythms to the environment. Trends Genet TIG.

[CR44] Lamaze A, Chen C, Leleux S (2022). ) A natural *timeless* polymorphism allowing circadian clock synchronization in ‘white nights’. Nat Commun.

[CR45] Lankinen P (1993). North-south differences in circadian eclosion rhythm in European populations of *Drosophila subobscura*. Hered Int J Genet.

[CR46] Lankinen P, Kastally C, Hoikkala A (2021). Nanda-Hamner curves show huge latitudinal variation but no circadian components in *Drosophila montana* photoperiodism. J Biol Rhythms.

[CR47] Lees AD (1960). Some aspects of animal photoperiodism. Cold Spring Harb Symp Quant Biol.

[CR48] Lees AD (1966). Photoperiodic timing mechanisms in insects. Nature.

[CR49] Lees AD (1973). Photoperiodic time measurement in the aphid *Megoura viciae*. J Insect Physiol.

[CR50] Lumme J, Lakovaara S, Ashburner M, Carson HL, Thompson JN (1983). Seasonality and diapause in Drosophilids. Genetics and biology of *Drosophila*.

[CR51] Lumme J, Oikarinen A, Lakovaara S, Alatalo R (1974). The environmental regulation of adult diapause in *Drosophila littoralis*. J Insect Physiol.

[CR52] Mathias D, Jacky L, Bradshaw WE, Holzapfel CM (2005). Geographic and developmental variation in expression of the circadian rhythm gene, *timeless*, in the pitcher-plant mosquito, *Wyeomyia smithii*. J Insect Physiol.

[CR53] Matsuda N (2023). Hatching rhythm and clock gene expression in the egg of the pea aphid, *Acyrthosiphon*
*pisum*. J Insect Physiol.

[CR54] Menegazzi P, Vanin S, Yoshii T (2013). *Drosophila* clock neurons under natural conditions. J Biol Rhythms.

[CR55] Menegazzi P, Dalla Benetta E, Beauchamp M (2017). Adaptation of circadian neuronal network to photoperiod in high-latitude European drosophilids. Curr Biol.

[CR56] Meuti ME, Stone M, Ikeno T, Denlinger DL (2015). Functional circadian clock genes are essential for the overwintering diapause of the Northern house mosquito, *Culex pipiens*. J Exp Biol.

[CR57] Nagy D, Cusumano P, Andreatta G (2019). Peptidergic signaling from clock neurons regulates reproductive dormancy in *Drosophila melanogaster*. PLoS Genet.

[CR58] Nanda KK, Hamner KC (1958). Studies on the nature of the endogenous rhythm affecting photoperiodic response of Biloxi Soybean. Bot Gaz.

[CR59] Numata H, Shiga S (1995). Induction of adult diapause by photoperiod and temperature in *Protophormia terraenovae* (Diptera: Calliphoridae) in Japan. Environ Entomol.

[CR60] Ojima N, Hara Y, Ito H, Yamamoto D (2018). Genetic dissection of stress-induced reproductive arrest in *Drosophila melanogaster* females. PLoS Genet.

[CR61] Pegoraro M, Zonato V, Tyler ER (2017). Geographical analysis of diapause inducibility in European *Drosophila melanogaster* populations. J Insect Physiol.

[CR62] Pittendrigh CS (1960). Circadian rhythms and the circadian organization of living systems. Cold Spring Harb Symp Quant Biol.

[CR63] Pittendrigh CS (1966). The circadian oscillation in *Drosophila pseudoobscura* pupae: a model for the photoperiodic clock. Z Pflanzenphysiol.

[CR64] Pittendrigh CS (1972). Circadian surfaces and the diversity of possible roles of circadian organization in photoperiodic induction. Proc Natl Acad Sci USA.

[CR65] Pittendrigh CS, Minis DH (1964). The entrainment of circadian oscillations by light and their role as photoperiodic clocks. Am Nat.

[CR66] Rieger D, Shafer OT, Tomioka K, Helfrich-Förster C (2006). Functional analysis of circadian pacemaker neurons in *Drosophila melanogaster*. J Neurosci.

[CR67] Rieger D, Peschel N, Dusik V (2012). The ability to entrain to long photoperiods differs between 3 *Drosophila melanogaster* wild-type strains and is modified by twilight simulation. J Biol Rhythms.

[CR68] Roenneberg T, Daan S, Merrow M (2003). The art of entrainment. J Biol Rhythms.

[CR69] Roenneberg T, Dragovic Z, Merrow M (2005). Demasking biological oscillators: properties and principles of entrainment exemplified by the *Neurospora* circadian clock. Proc Natl Acad Sci USA.

[CR70] Salminen TS, Vesala L, Laiho A (2015). Seasonal gene expression kinetics between diapause phases in *Drosophila virilis* group species and overwintering differences between diapausing and non-diapausing females. Sci Rep.

[CR71] Sandrelli F, Tauber E, Pegoraro M (2007). A molecular basis for natural selection at the timeless locus in *Drosophila melanogaster*. Science.

[CR72] Saunders DS (1978). Internal and external coincidence and the apparent diversity of photoperiodic clocks in the insects. J Comp Physiol.

[CR73] Saunders DS (1990). The circadian basis of ovarian diapause regulation in *Drosophila melanogaster*: is the period gene causally involved in photoperiodic time measurement?. J Biol Rhythms.

[CR74] Saunders DS (2020). Dormancy, diapause, and the role of the circadian system in insect photoperiodism. Annu Rev Entomol.

[CR75] Saunders DS (2021). A comparative study of circadian rhythmicity and photoperiodism in closely related species of blow flies: external coincidence, maternal induction, and diapause at northern latitudes. J Biol Rhythms.

[CR76] Saunders DS (this issue) Time measurement in insect photoperiodism: External and internal coincidence. J Comp Physiol A 10.1007/s00359-023-01648-4 PMC11226529 37697123

[CR77] Saunders DS, Lewis RD (1987). A damped circadian oscillator model of an insect photoperiodic clock: III. Circadian and “hourglass” responses. J Theor Biol.

[CR78] Saunders DS, Henrich VC, Gilbert LI (1989). Induction of diapause in *Drosophila melanogaster*: photoperiodic regulation and the impact of arrhythmic clock mutations on time measurement. Proc Natl Acad Sci USA.

[CR79] Schlichting M, Helfrich-Förster C (2015). Photic entrainment in *Drosophila* assessed by locomotor activity recordings. Methods Enzymol.

[CR80] Schmid B, Helfrich-Förster C, Yoshii T (2011). A new ImageJ plug-in “ActogramJ” for chronobiological analyses. J Biol Rhythms.

[CR81] Sehgal A, Price JL, Man B, Young MW (1994). Loss of circadian behavioral rhythms and per RNA oscillations in the *Drosophila* mutant *timeless*. Science.

[CR82] Shafer OT, Levine JD, Truman JW, Hall JC (2004). Flies by night: effects of changing day length on *Drosophila* circadian clock. Curr Biol.

[CR83] Shiga S, Numata H (2009). Roles of PER immunoreactive neurons in circadian rhythms and photoperiodism in the blow fly, *Protophormia terraenovae*. J Exp Biol.

[CR84] Sim C, Denlinger DL (2008). Insulin signaling and FOXO regulate the overwintering diapause of the mosquito *Culex pipiens*. Proc Natl Acad Sci USA.

[CR85] Sim C, Denlinger DL (2013). Insulin signaling and the regulation of insect diapause. Front Physiol.

[CR86] Stanewsky R, Kaneko M, Emery P (1998). The *cryb* mutation identifies cryptochrome as a circadian photoreceptor in *Drosophila*. Cell.

[CR87] Stehlík J, Závodská R, Shimada K (2008). Photoperiodic induction of diapause requires regulated transcription of *timeless* in the larval brain of *Chymomyza costata*. J Biol Rhythms.

[CR88] Tauber E, Zordan M, Sandrelli F (2007). Natural selection favors a newly derived *timeless* allele in *Drosophila melanogaster*. Science.

[CR89] Vanin S, Bhutani S, Montelli S (2012). Unexpected features of *Drosophila* circadian behavioural rhythms under natural conditions. Nature.

[CR90] Vaze KM, Helfrich-Förster C (2016). *Drosophila ezoana* uses an hour-glass or highly damped circadian clock for measuring night length and inducing diapause. Physiol Entomol.

[CR91] Vaze KM, Helfrich-Förster C (2021). The neuropeptide PDF is crucial for delaying the phase of *Drosophila’s* evening neurons under long Zeitgeber periods. J Biol Rhythms.

[CR96] Wickham H (2016) Ggplot2: Elegant graphics for data analysis. Springer-Verlag, New York. https://ggplot2-book.org/

[CR92] Yamada H, Yamamoto M-T (2011). Association between circadian clock genes and diapause incidence in *Drosophila triauraria*. Plos one.

[CR93] Yoshida T, Kimura MT (1995). The photoperiodic clock in *Chymomyza costata*. J Insect Physiol.

[CR94] Yoshii T, Todo T, Wülbeck C (2008). Cryptochrome is present in the compound eyes and a subset of *Drosophila’s* clock neurons. J Comp Neurol.

[CR95] Yoshii T, Rieger D, Helfrich-Förster C (2012). Two clocks in the brain: an update of the morning and evening oscillator model in *Drosophila*. Prog Brain Res.

