# Supplementary material for: Drosophila ezoana uses morning and evening oscillators to adjust its rhythmic activity to different daylengths but only the morning oscillator to measure night length for photoperiodic responses
Source: J Comp Physiol A Neuroethol Sens Neural Behav Physiol. 2023 Jun 17;210(4):535–48. doi: 10.1007/s00359-023-01646-6 (PMC11226516; doi:10.1007/s00359-023-01646-6)
Supplement: Supplementary file 1 — Supplementary file1 (DOCX 283 KB) [file 359_2023_1646_MOESM1_ESM.docx]

**Supplementary Material**


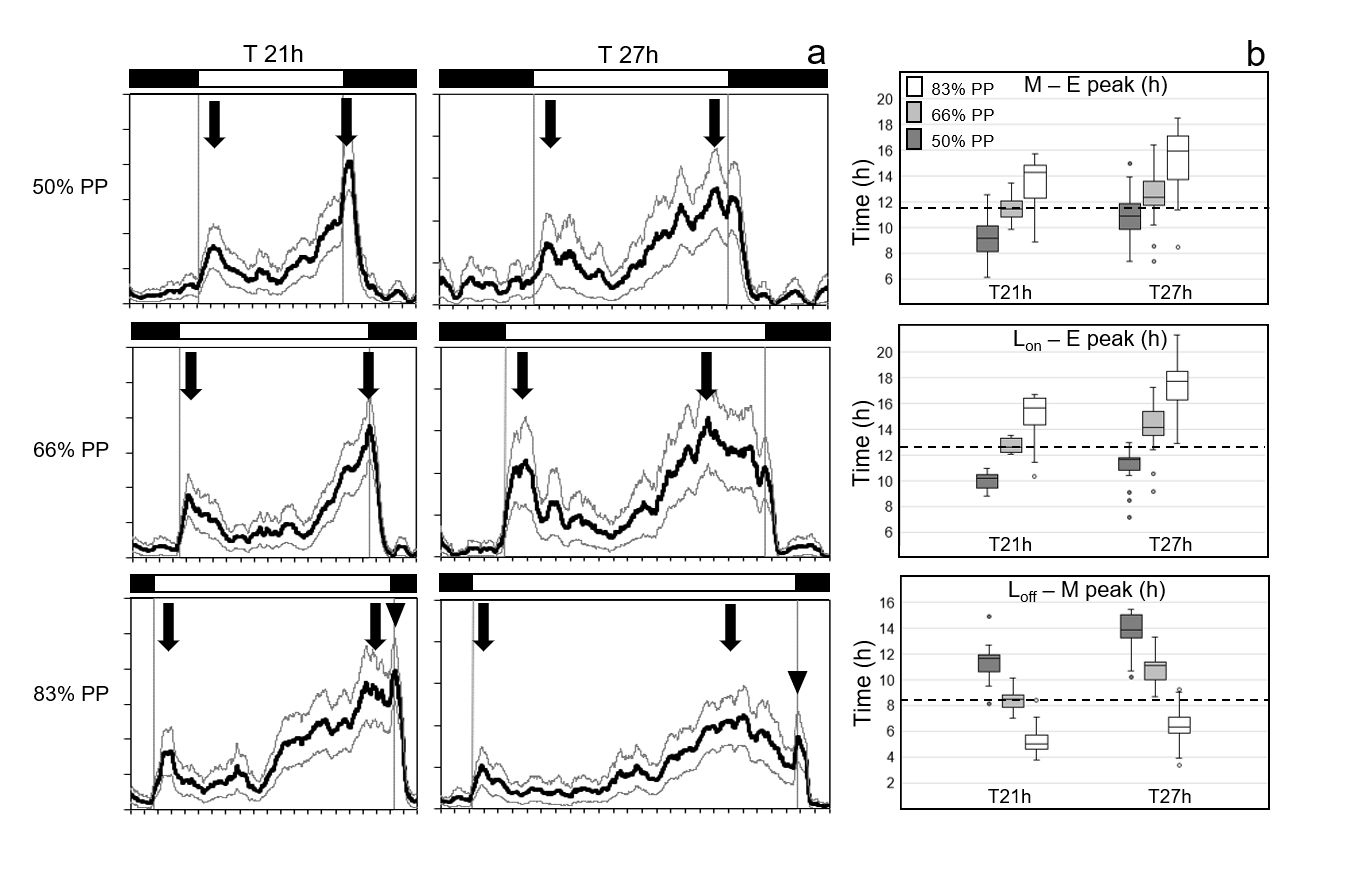


**Fig. S1** Activity data for male *D. ezoana* flies. **a** Average activity profiles at the three photoperiods (PP) and two Zeitgeber periods (T). Thick arrows mark the estimated peaks of morning and evening activity, while arrowheads mark the rare visible lights-on and lights-off startle responses. **b** Boxplots showing the time passed between morning and evening peaks (M – E peak), between lights-on and the evening peak (L_on_-E peak), and between lights-off and the morning peak (L_off_-M peak) at the 3 photoperiods and 2 Zeitgeber periods. The horizontal stippled lines represent the critical nightlength (c) for diapause induction as determined by Vaze and Helfrich-Förster (2016).
