# Supplementary figures and images for: Drosophila ezoana uses morning and evening oscillators to adjust its rhythmic activity to different daylengths but only the morning oscillator to measure night length for photoperiodic responses
Source: J Comp Physiol A Neuroethol Sens Neural Behav Physiol. 2023 Jun 17;210(4):535–48. doi: 10.1007/s00359-023-01646-6 (PMC11226516; doi:10.1007/s00359-023-01646-6)

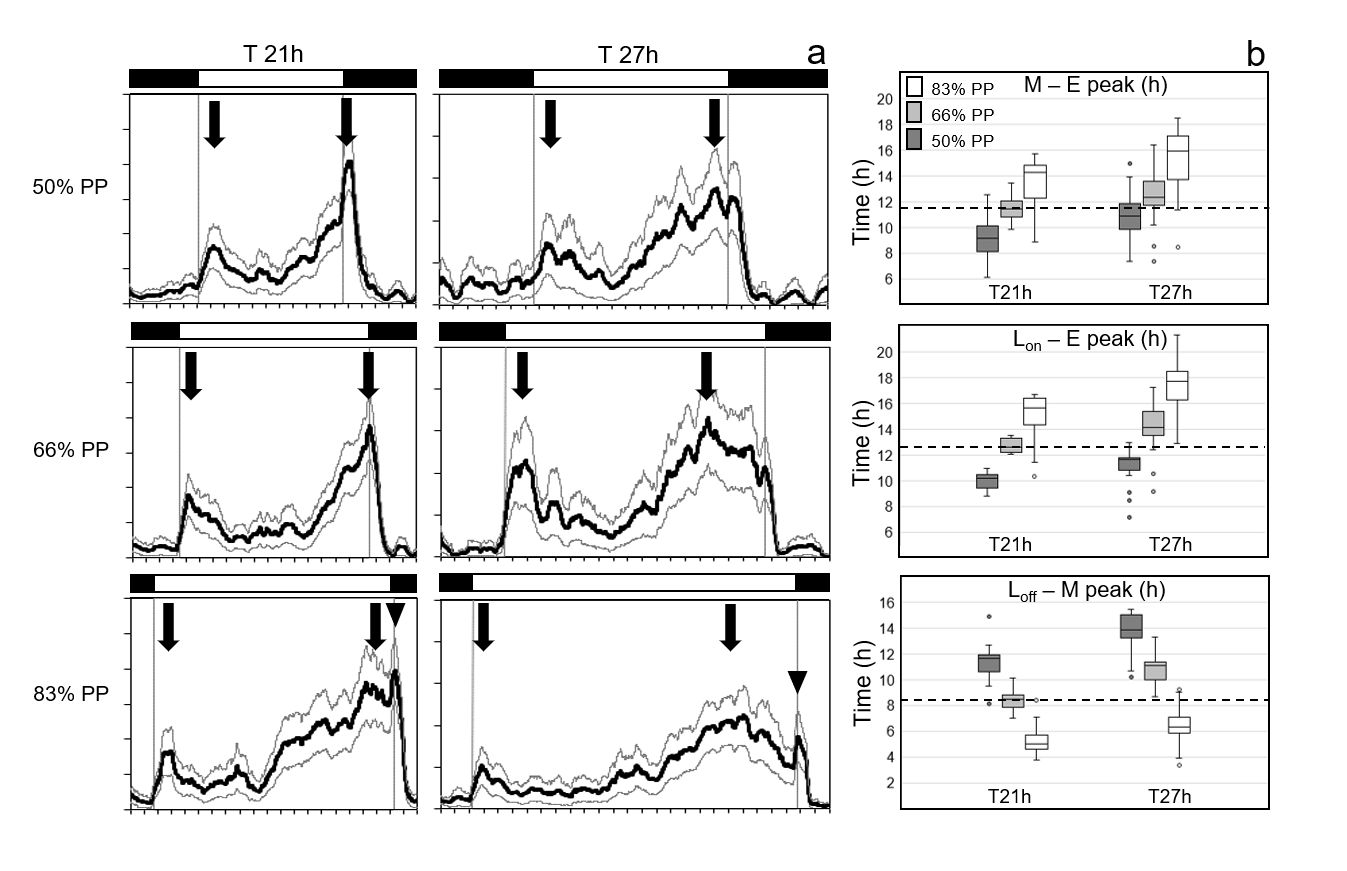

Supplement: Supplementary file 2 — Supplementary file2 (TIF 270 KB) [file 359_2023_1646_MOESM2_ESM.tif]
